# Transcriptomic changes in peripheral blood mononuclear cells with weight loss: systematic literature review and primary data synthesis

**DOI:** 10.1186/s12263-021-00692-6

**Published:** 2021-07-19

**Authors:** Kaitlin Day, Aimee L. Dordevic, Helen Truby, Melissa C. Southey, Susan Coort, Chiara Murgia

**Affiliations:** 1 Department of Nutrition, Dietetics and Food, Monash University, Level 1, 264 Ferntree Gully Road, Notting Hill, Victoria 3168 Australia; 2 School of Human Movement and Nutrition Sciences, University of Queensland, Brisbane, Australia; 3 Department of Clinical Pathology, Melbourne Medical School, The University of Melbourne, Melbourne, Victoria Australia; 4 Cancer Epidemiology Division, Cancer Council Victoria, Melbourne, Victoria Australia; 5 Precision Medicine, School of Clinical Sciences at Monash Health, Monash University, Clayton, Victoria Australia; 6 Department of Bioinformatics-BiGCaT, NUTRIM School of Nutrition and Translational Research in Metabolism, Maastricht University, Maastricht, The Netherlands; 7 School of Agriculture and Food, The University of Melbourne, Melbourne, Australia

**Keywords:** Transcriptomics, Obesity, Weight loss intervention, Overweight, Gene expression

## Abstract

**Background and objectives:**

Peripheral blood mononuclear cells (PBMCs) have shown promise as a tissue sensitive to subtle and possibly systemic transcriptomic changes, and as such may be useful in identifying responses to weight loss interventions. The primary aim was to comprehensively evaluate the transcriptomic changes that may occur during weight loss and to determine if there is a consistent response across intervention types in human populations of all ages.

**Methods:**

Included studies were randomised control trials or cohort studies that administered an intervention primarily designed to decrease weight in any overweight or obese human population. A systematic search of the literature was conducted to obtain studies and gene expression databases were interrogated to locate corresponding transcriptomic datasets. Datasets were normalised using the ArrayAnalysis online tool and differential gene expression was determined using the limma package in R. Over-represented pathways were explored using the PathVisio software. Heatmaps and hierarchical clustering were utilised to visualise gene expression.

**Results:**

Seven papers met the inclusion criteria, five of which had raw gene expression data available. Of these, three could be grouped into high responders (HR, ≥ 5% body weight loss) and low responders (LR). No genes were consistently differentially expressed between high and low responders across studies. Adolescents had the largest transcriptomic response to weight loss followed by adults who underwent bariatric surgery. Seven pathways were altered in two out of four studies following the intervention and the pathway ‘cytoplasmic ribosomal proteins’ (WikiPathways: WP477) was altered between HR and LR at baseline in the two datasets with both groups. Pathways related to ‘toll-like receptor signalling’ were altered in HR response to the weight loss intervention in two out of three datasets.

**Conclusions:**

Transcriptomic changes in PBMCs do occur in response to weight change. Transparent and standardised data reporting is needed to realise the potential of transcriptomics for investigating phenotypic features.

**Registration number:**

PROSPERO: CRD42019106582

**Supplementary Information:**

The online version contains supplementary material available at 10.1186/s12263-021-00692-6.

## Background

There has been a global increase in the prevalence of obesity over the last 40 years. This rise has been challenging to abate, despite the development of a variety of specific interventions at the individual level and attempts to shift food and exercise patterns at the public health level [1]. A 2007 systematic review and meta-analysis that assessed weight outcomes of 26,455 participants across eight different intervention types reported high heterogeneity, in relation to efficacy, across all intervention types and approaches with mean weight change ranging from + 0.7 kg to – 22.0 kg after 6 months of treatment [2]. A recent meta-analysis of genome-wide association studies, to understand the genetic determinants of BMI, suggested genetic variation accounts for 24.6% of the variation in BMI and combined this evidence supports the hypothesis that an individual’s weight and weight loss response is complex and highly variable [3]. It is now accepted that ‘one size does not fit all’ in terms of weight loss approaches.

Our lack of understanding of how subtle differences in physiology could play a role in treatment response is a barrier to personalising approaches that would optimise an individual’s outcome [4]. It is well established that the location of body fat determines disease risk, due to the relationship between adipose tissue type and its endocrine activity [5, 6]. Global gene expression levels, in specific tissues, are highly sensitive to endogenous and exogenous stimuli and as such, can provide insight into mechanistic and often subtle differences in individuals [7]. Individual gene expression response may be predictive of success, as shown by distinctive gene expression patterns in subcutaneous adipose tissue in weight loss maintainers compared with participants who rapidly regained weight lost through a weight loss intervention [8].

Peripheral blood mononuclear cells (PBMCs) have shown promise as a tissue of exploration in obesity research as they are exposed to a range of metabolites from the diet and resulting from physiological changes in multiple tissues [9]. Due to their ease of collection from blood, PBMCs offer the opportunity to measure valuable information about the metabolic response to the intervention. Both the questions of whether (i) information obtained from PBMCs can help stratify individuals and (ii) if this can go beyond the simple measurement of quantity (volume) of weight or body fat lost remain unanswered.

This review sought to explore global gene expression changes in PBMCs before and after a weight loss intervention in human populations of all ages. This review considered randomised control trials and cohort studies that administered an intervention primarily designed to decrease weight in any human population with overweight or obesity. The primary aim was to comprehensively evaluate the transcriptomic changes that may occur during weight loss and to determine if there is a systematic response across intervention types. Secondly, by locating primary data, we aimed to assess the gene expression differences between participants who respond differently to the intervention (high versus low weight loss) to elucidate any potential patterns of transcriptomic response that differ between high and low responders.

## Methods

This review was prospectively registered with PROSPERO (No. CRD42019106582).

### Search Strategy

A literature search was conducted in July 2019 with no date limits*.* Table 1 contains the search strategy adapted for use in OVID Medline, Embase, Cochrane, Cinahl, Scopus and Web of Science; a combination of MeSH terms and free-text searches were used. Literature cited in relevant papers were also assessed for eligible articles. Available datasets were retrieved from the online repositories GEO [10] and ArrayExpress [11].

**Table 1:** Search terms

Query number	Search term
1	overweight/ or exp obesity/[MeSH]
2	(overweight or over weight).mp.
3	obes*.mp.
4	Adiposity/[MeSH]
5	adipos*.mp.
6	1 or 2 or 3 or 4 or 5
7	((weight or exercise or lifestyle or life style or diet* or food* or intake or nutrition* or resistance or physical or aerobic or strength) adj5 (intervention* or program* or therap* or training or trial* or counsel* or educ*)).mp.
8	exp Nutrition Therapy/[MeSH]
9	bariatric surger*.mp.
10	exp Bariatric Surgery/[MeSH]
11	Obesity Management/[MeSH]
12	7 or 8 or 9 or 10 or 11
13	gene expression.mp.
14	gene expression/ or transcription, genetic/ or transcriptome/[MeSH]
15	transcriptom*.mp.
16	gene* transcript*.mp.
17	rna/ or rna, messenger/[MeSH]
18	Rna.mp.
19	microarray*.mp.
20	exp Microarray Analysis/ OR sequencing, RNA/[MeSH]
21	nutrigenomics.mp.
22	13 or 14 or 15 or 16 or 17 or 18 or 19 or 20 or 21
23	6 and 12 and 22
24	limit 23 to humans

Search terms were adapted for use in OVID Medline, Embase, Cochrane, Cinahl, Scopus and Web of Science databases. Mp: title, abstract, original title, name of substance word, subject heading word, floating sub-heading word, keyword heading word, protocol supplementary concept word, rare disease supplementary concept word, unique identifier, synonyms

### Selection of studies and data extraction

#### Inclusion criteria

Studies were included that reported original research conducted in humans of any age, classified as having overweight or obesity (defined by BMI > 25 kg/m^2^ or equivalent). The interventions must have included a weight loss component and measured global gene expression (either through microarray technology or RNA sequencing) in PBMCs at baseline and after the intervention as an outcome. Inclusion of a control group was not required.

#### Exclusion criteria

Studies were excluded if they did not involve human participants or if participants were pregnant at the time of intervention. Studies that were not published in English or not presented in an original research communication, e.g. conference proceedings, single case studies or book chapters were also excluded.

### Data collection

#### Selection of studies

Two reviewers independently screened titles and abstracts of studies retrieved from the search strategy. Full-text articles were retrieved for selected studies and were independently screened against the inclusion and exclusion criteria by the two reviewers and conflicts were discussed and resolved by the group.

#### Data extraction

A template extraction table was created to capture information regarding the study design, intervention design, methods of RNA isolation, weight outcomes, gene expression analysis and other relevant outcomes. Two reviewers independently extracted data from the included studies and cross-checked to verify findings. Any discrepancies were resolved through discussion. Corresponding authors were contacted to obtain any missing information.

#### Quality assessment of the studies

A tool previously created by the authors was adapted for use in the current review [12]. The tool was adapted by replacing the domain assessing genetic variants with a domain assessing appropriate experimental control and filtering of expression data (Additional file 1).

### Quantitative data analysis

**Fig. 1:**
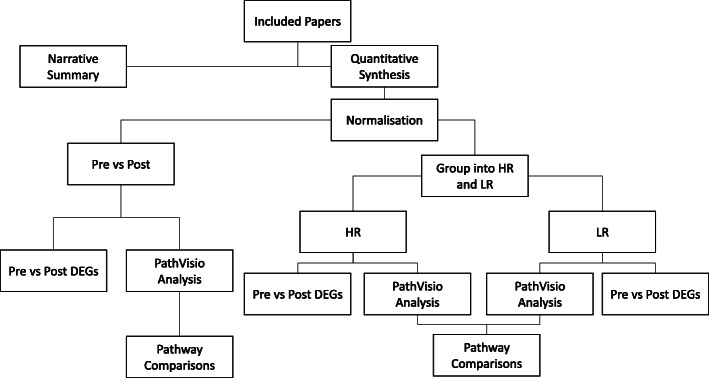
Workflow of methods of analysis of included papers after literature search. Where no raw or normalised global gene expression data was available, papers were summarised narratively. Normalisation was conducted using the ArrayAnalysis online workflow (available at: arrayanalysis.org) according to the microarray chip type. Where only normalised global gene expression data was available, the data entered the pipeline after the normalisation step. High responders were defined as those that lost ≥ 5% body weight over the intervention period. Comparisons between included papers were made at the pathway level. DEGs, differentially expressed genes, defined as any gene with an adjusted *p* value < 0.05; HR, high responder; LR, low responder

As all included studies utilised microarray technology, the quantitative analysis of all studies followed the same workflow previously described by A Muñoz Garcia et al. [13]. This workflow allows for the integration of multiple global gene expression datasets and places them in a biological context (see Fig. 1 for an overview of the methods [13]).

#### Data inclusion criteria

The raw sample-level gene expression data were obtained from online repositories (GEO and ArrayExpress). Where datasets were not publicly available, the corresponding authors were contacted to provide the raw data. If the raw gene expression data were not available, normalised individual level gene expression data were obtained.

Studies were excluded from the quantitative synthesis if they had < 50% of the sample-level gene expression data available as either raw gene expression data or normalised, or were completed > ten years ago.

Authors were contacted to provide individual-level data relating to weight outcomes so that individuals could be re-grouped into HR and LR to each intervention. HR were defined as individuals who lost ≥ 5% body weight over the intervention period [14], and low responders were those that lost < 5% body weight over the intervention period.

#### Quality control and normalisation of raw gene expression data

The available raw microarray data underwent quality control checks and were normalised using ArrayAnalysis, a standardised pipeline [15]. The most appropriate normalisation method was selected by the analysis pipeline, this was determined to be robust multi-array averaging (RMA) for all included datasets.

#### Determining differentially expressed transcripts

Significantly differentially expressed transcripts were determined by linear modelling of normalised data using the limma package [16] in R [17] (R version 3.6.1., limma version 3.40.6), and Benjamini-Hochberg correction for multiple testing [18]. Transcripts were defined as significantly differentially expressed with an adjusted *p* value < 0.05. Paired analysis was utilised to compare baseline and post-intervention gene expression levels within participants and unpaired analysis was utilised to compare baseline HR and LR gene expression levels.

Significantly differentially expressed genes were visualised using heatmaps and hierarchical clustering performed using the gplots package in R (R version 3.6.1, gplots version 3.0.1.1.) using Euclidean distance for clustering.

#### Pathway analysis

Overrepresented pathways were identified using the PathVisio software (version 3.3.0) which utilises the Wikipathways database [19, 20]. The human pathways were used for this analysis (downloaded: 12/04/2019) which contains 521 distinct pathways. Pathways were considered overrepresented if they met the following criteria: *z*-score ≥ 1.96 and at least five genes within the pathway had an unadjusted *p* value < 0.05 [13]. For pathways that were overrepresented in multiple studies, log fold changes of genes within the pathway were visualised using heatmaps and hierarchical clustering performed as above. Where there were multiple probes associated with a given gene, the log fold changes of the probes were averaged together to give a single value per gene within the pathway. These heatmaps were used for visualisation purposes only.

## Results

### Study characteristics

**Fig. 2:**
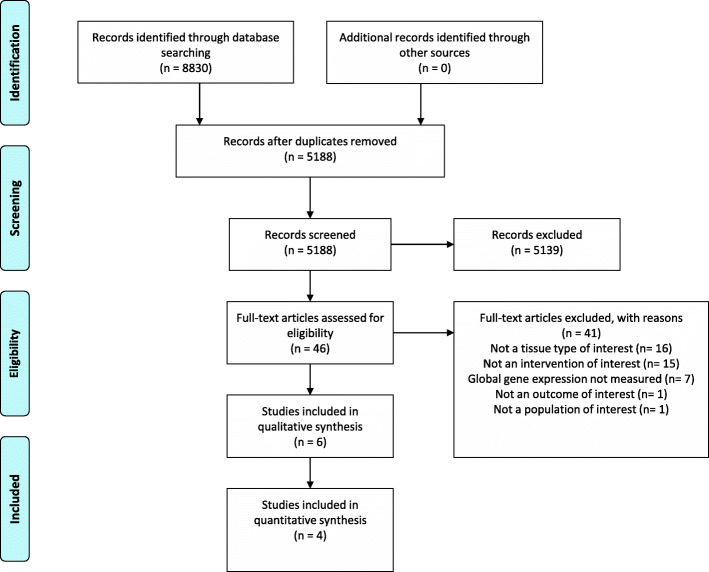
PRISMA flow diagram of studies included in the systematic review

The search was conducted in July 2019 and 5329 studies were included in the first-pass screening after the removal of duplicates. After full-text screening, seven articles, pertaining to six studies, met inclusion criteria **(**Fig. 2) [21–27]. The main reasons articles were excluded were due to studies assessing global gene expression in whole blood rather than PBMCs or the intervention did not contain a weight loss component (e.g. participants were instructed to remain weight stable throughout the intervention period, see additional file 2 for full exclusion list). The six included studies represented 118 participants who underwent a weight loss intervention. Table 2 describes the studies’ characteristics. Two studies were randomised control trials [25, 26] and four studies were cohort studies [21–24, 27]. One study was conducted in twelve adolescent males [24], the other five were conducted in adults in which two studies’ participants were all male [21, 26], two studies’ participants were all female [22, 23] and, for one, the cohort comprised of both male and female participants [25]. In one study, the intervention was Roux-en-Y gastric bypass bariatric surgery [23, 27]; for all others, the intervention was focused around reduction in dietary energy. The longest intervention duration was 6 months [25] and the shortest duration was four to five weeks (one full menstrual cycle) [22]. Mean weight loss across the studies ranged from 3.3–28.8 kg. The greatest weight loss occurred in adults who underwent bariatric surgery [23, 27], and the lowest was in females who underwent intermittent energy restriction for one full menstrual cycle [22]. All included articles measured global gene expression using microarray technology.

**Table 2:** Study characteristics of included studies

Study	Study design	Sample size	Population^a^	Intervention duration	Intervention description	RNA extraction	Microarray	Weight change^a^	Transcript response^b, c^		Pathway response^b, d^	
									Effect of intervention	HR vs LR	Effect of intervention	HR vs LR
Crujeiras et al. 2008 [21]	Cohort	9	44.8 ± 5.2 year, BMI: 44.8 (5.2) kg/m^2^, 100% male, 100% Caucasian	8 weeks	35% energy restriction, 55% carbohydrates, 30% lipids, 15% protein	Trizol	Agilent Human 1A Oligo V2	− 8.9 ± 1.5 %	158 increased 227 decreased^e^	NA	NA	7^e^
Harvie et al. 2016 [22]	Cohort	20 (HR = 7)	40.3 ± 3.2 years, BMI: 28.3 (3.1) kg/m^2^, 100% female, 96% Caucasian	4–5 weeks (1 full menstrual cycle)	Overall 25% energy restriction with 2 consecutive days 65% energy restriction, 5 days no restriction, restriction days—100 g carbohydrates and 50 g protein, non-restricted days—45% low-GI carbohydrates, 30% lipids, 25% protein	RNeasy plus kit	Affymetrix U133 plus2	− 3.3 kg	0	0	8	20
Pinhel et al. 2017 [23] and Pinhel et al. 2018 [27]	Cohort	13	39 ± 9 years, BMI: 42.5 (7.0) kg/m^2^, 100% female, ethnicity NA	6 months	Roux-en-Y gastric bypass surgery	Phenol/chlorform	Illumina HumanHT-12 V4	− 28.8 kg	7 increased 21 decreased	NA	13	NA
Rendo-Urteaga et al. 2015 [24]	Cohort	12 (HR = 6)	13.17 ± 0.98^f^ years, BMI-SDS: HR: 3.10 (0.54), LR 4.08 (0.43)^f^, 100% male, ethnicity NA	10 weeks	Between 1300 and 9204 kcal/day calculated based on energy requirements and severity of obesity	Trizol	Affymetrix Human 1.1 ST	HR: − 4.47 kg (0.23)^f^ LR: + 0.75 kg	372 increased 456 decreased	0 increased in HR 23 decreased in HR	53	21
Samblas et al. 2018 [25]	Randomised control trial^g^	Discovery = 24, validation = 37	48.4 ± 3.5 years, BMI: discovery: HR: 34.9 (3.0) kg/m^2^, LR: 37.0 (2.1) kg/m^2^, validation: HR: 33.8 (3.6) kg/m^2^ LR: 36.1 (5.0) kg/m^2^, 54% male, ethnicity NA	6 months	30% energy restriction, 40% carbohydrates, 30% lipids, 30% protein	Trizol	Illumina HumanHT-12 V4	Discovery: HR: − 10.5% ± 1.8%, LR: − 5.9% ± 1.7% Validation: HR: − 9.5% ± 2.0%, LR: − 5.7% ± 2.1%	NA	20 increased in HR 136 decreased in HR^e^	NA	NA
van Bussel et al. 2019 [26]	Randomised Control Trial	40	60 (50–65)^h^ years, BMI: 29.2 (3.0) kg/m^2^, 52% male, 100% Caucasian	12 weeks	20% energy restriction, 50% carbohydrates, 35% lipids, 15% protein	Trizol	Affymetrix Human 1.1 ST	− 5.6 ± 2.9 kg	0	NA	15	NA

^a^Results are presented as mean (SD) unless otherwise stated

^b^results from quantitative analysis conducted as part of this review unless otherwise stated

^c^transcriptomic response describes transcripts that were significantly differentially expressed defined as an absolute fold change > 1.2 or < − 1.2 and adjusted *p* value < 0.05

^d^pathway response describes wikipathways that were overrepresented defined as pathways with a *z*-score < 1.96 and 5 or more genes in the pathway with an unadjusted *p* value < 0.05

^e^data reported in article

^f^data presented as mean (standard error of the mean)

^g^results of intervention and control pooled for transcriptomic analysis

^h^ results presented as mean (range). *HR* high responders defined as > 5% body weight lost over the intervention period, *LR* low responders, *NA* not available, *BMI-SDS* BMI standard deviation score

### Quality assessment

Four of the seven articles included were assessed as positive [22, 24–26], two as neutral [23, 27] and one as negative [21] for overall quality and risk of bias (Additional file 1). Studies that were assigned negative or neutral generally did not describe quality control procedures with regard to RNA extraction and expression data in sufficient detail.

### Summary of included studies

Of the seven included articles, six reported significant changes in PBMC gene expression levels in response to a weight loss intervention [21, 23–27] and one reported no change [22]. Of the six studies reporting significant gene expression changes, five performed further functional analysis to explore enriched gene ontology and/or biological pathways [21, 23, 24, 26, 27]. All studies reporting significant gene expression changes reported overrepresentation of genes in pathways related to gene expression regulation and immune signalling. Crujeiras et al. [21] and van Bussel et al. [26] identified that transcripts associated with a number of oxidative stress and inflammation pathways responded to an 8-week 35% energy reduction or a 20% energy restriction diet for 12 weeks respectively. In particular, transcripts for the genes *TANK* and *TRAID3* were upregulated after weight loss in Crujeiras et al.; both genes are involved in the NF-κB signalling pathway which is a known immune modulation pathway [21, 28]. Samblas et al. identified CD44 as a potential biomarker of weight loss, finding that *CD44* was upregulated and hypomethylated in LR compared to HR [25]. Pinhel et al., the only study in bariatric surgery [23, 27], identified differential expression of genes controlling gene expression regulatory and signalling mechanisms, including the mTOR pathway, genes related to translation and TLR4 signalling, post-operatively compared to pre-operatively. Rendo-Urteaga et al. [24], the only study to explore gene expression changes in response to weight loss in adolescents reported differences in HR and LR at baseline, in particular, decreased expression of genes involved in inflammatory processes (including *LEPR* and *SIRPB1*) and pathways related to cardiomyopathy in HR compared to LR. In this study, LR were defined as maintaining or increasing BMI standard deviation score (BMI SDS) over the intervention period. Harvie et al. [22] had the shortest duration intervention at one full menstrual cycle (four to five weeks) and reported no differences in gene expression levels in PBMCs in blood samples taken before and after the intervention period.

### Quantitative synthesis

After searching online data repositories and contacting authors, raw gene expression data was obtained for 3 studies [22, 24, 26] and normalised data was obtained for one study [23, 27]. Two authors provided individual weight data [22, 24]. One paper with available raw gene expression data was not included in the quantitative synthesis as less than 50% of the data was available and had been conducted more than ten years ago [21]. Normalised gene expression data were only available for one study, which had been normalised using average normalisation through GenomeStudio (Illumina, San Diego, California, United States) [23, 27]. Statistical analysis of differentially expressed transcripts and pathways were completed within studies and these results were compared and contrasted across studies due to differences in populations and study designs.

#### Effect of intervention on gene expression

After correction for multiple testing, no transcripts were significantly differentially expressed in PBMCs after the intervention in Harvie et al. [22] and van Bussel et al. [26] (adj *p* > 0.05). Of the two remaining datasets, 828 transcripts were significantly differentially expressed (adj *p* < 0.05) in PBMCs after the intervention in Rendo-Urteaga et al. [24] and 28 transcripts in Pinhel et al. (Additional file 3) [23, 27]. No genes or groups of genes were commonly differentially expressed across all studies.

**Fig. 3:**
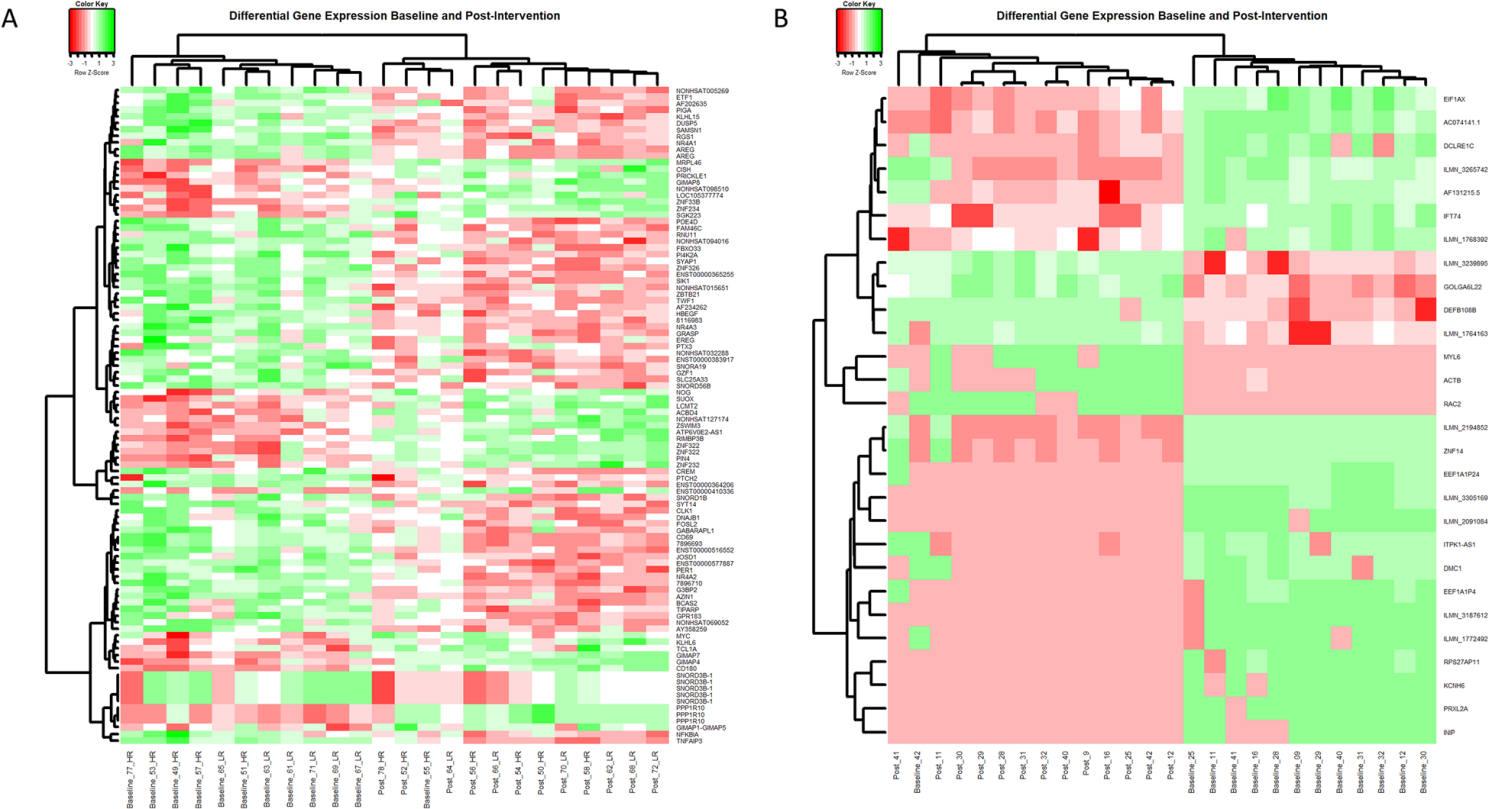
Heatmap and hierarchical clustering of the differentially expressed genes when comparing baseline to post-intervention samples. Heatmaps were created using the gplots package (version 3.0.1.1.) in R (version 3.6.1). Columns represent individual samples and rows represent individual genes. Samples have been clustered using Euclidean distancing. **A** Top 100 significantly differentiated genes for Rendo-Urteaga et al. (adj. *p* value <0.05). **B** The 28 significantly differentiated genes for Pinhel et al. (adj. *p* value <0.05)

Heatmaps and hierarchical clustering **(**Fig. 3) revealed a clear separation of gene expression between baseline and post-intervention samples in Rendo-Urteaga et al. and Pinhel et al., (Fig. 3 a and b) [23, 24].

Pathway analysis revealed that there was some overlap between studies (Additional file 4, table 1). However, there were no pathways common to all four explored studies. Fifty-three pathways were altered in PBMCs after the intervention in Rendo-Urteaga et al., fifteen in vanBussel et al., thirteen in Pinhel et al. and eight pathways were affected in Harvie et al. [22, 24, 26]. There were two pathways that were common to Rendo-Urteaga et al. and Pinhel et al., ‘miRNAs involved in DNA damage response’ (WikiPathways: WP1545) and ‘circadian rhythms related genes’ (WikiPathways: WP3594) [23, 24] (Additional file 4). Rendo-Urteaga et al. and vanBusel et al. had three overlapping pathways, ‘apoptosis modulation and signalling’ (WikiPathways: WP3624), ‘IL-4 signalling pathway’ (WikiPathways: WP395) and ‘B cell receptor signalling pathway’ (WikiPathways: WP23) [24, 26].

#### Comparison of the global gene expression response of HR and LR to a weight loss intervention

Two articles provided individual-level weight change data and individual subjects were then grouped into high (*n* = 13) and low (*n* = 17) responders (Harvie et al. and Rendo-Urteaga et al.) [22, 24]. All participants in Pinhel et al. had significant weight loss 6 months after bariatric surgery, and so, the whole group were deemed HR (baseline weight: 115.3 ± 19.4 kg, follow-up weight: 85.3 ± 13.8 kg, *n* = 13) [23].

#### Comparison of gene expression levels between HR and LR at baseline

**Fig. 4:**
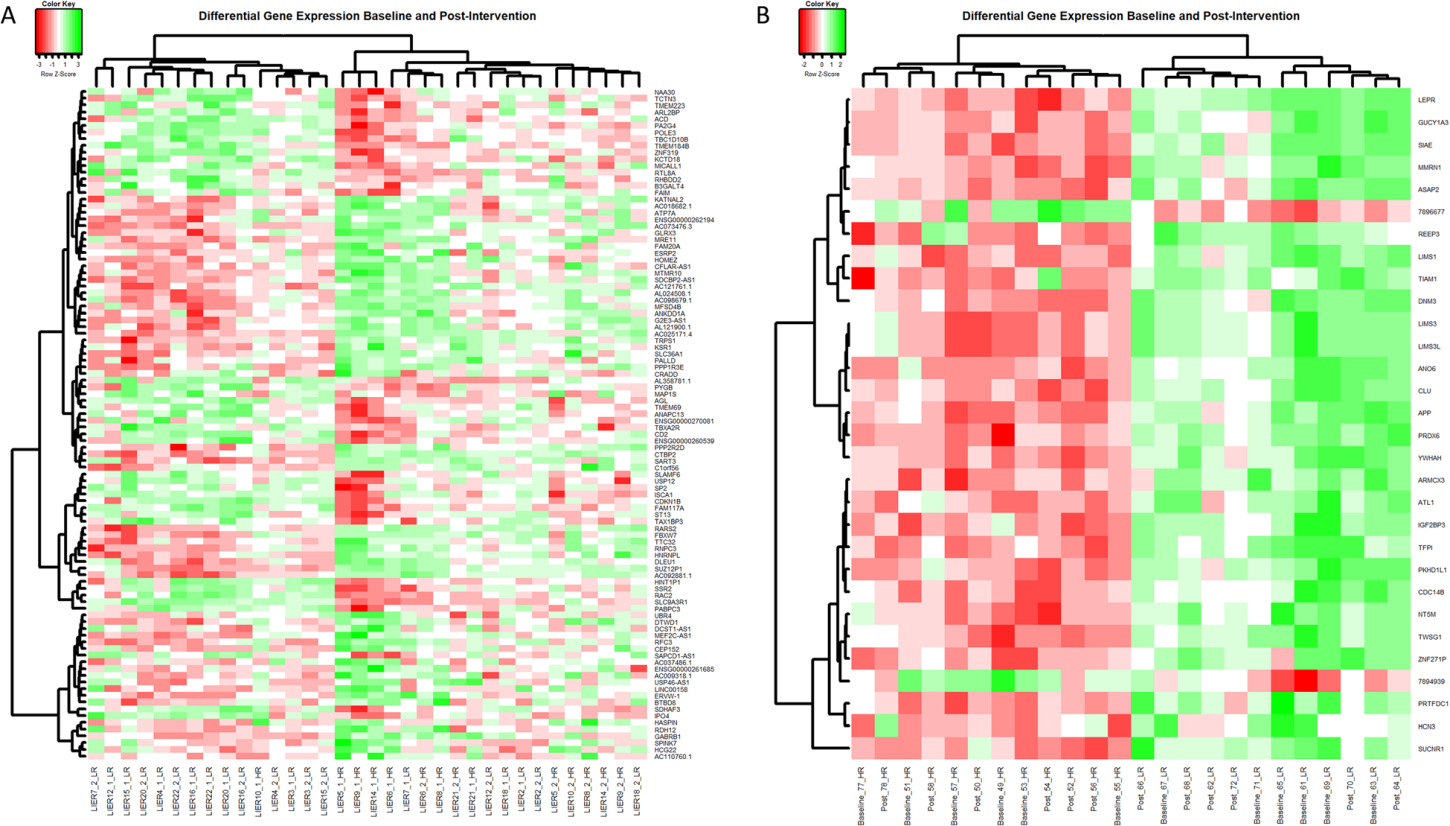
Heatmap and hierarchical clustering of the differentially expressed genes when comparing HR and LR samples at baseline. Heatmaps were created using the gplots package (version 3.0.1.1.) in R (version 3.6.1). Columns represent individual samples and rows represent individual genes. Samples have been clustered using Euclidean distancing. **A** Top 100 differentially expressed (*p* value > 0.05) between HR and LR at baseline for Harvie et al. **B** The 30 significantly differentially expressed genes (adj. *p* value < 0.05) between HR and LR at baseline for Rendo-Urteaga et al. HR, high responders defined at those that lost > 5% body weight over the intervention period. LR, low responders

For Rendo-Urteaga et al. [24], 23 transcripts were significantly differentially expressed between HR and LR at baseline (Fig. 4a, Additional file 3). *LEPR* (Leptin Receptor) expression levels were lower in PBMCs of HR compared with LR (log fold change − 0.43, adj. *p* value <0.001). There were no significantly differentially expressed transcripts (adjusted *p* value < 0.05) between HR and LR at baseline for Harvie et al. [22]. Pathway analysis of Rendo-Urteaga et al. found that 21 pathways differed between HR and LR and 20 pathways altered in Harvie et al. between HR and LR at baseline (Additional file 4, table 2) [22, 24]. Two pathways were common between the two studies, ‘cytoplasmic ribosomal proteins’ (WikiPathways: WP477, Additional file 6) and ‘Initiation of transcription and translation elongation at the HIV-1 LTR’ (Wikipathways: WP3414).

Heatmaps and hierarchical clustering showed a separation of HR and LR samples in both studies despite no transcripts being significantly differentially expressed (adjusted *p* value < 0.05) between HR and LR in Harvie et al. (Fig. 4b) [22]. In Rendo-Urteaga et al., all but two of the 23 significantly differentially expressed transcripts were downregulated in HR compared to LR (Additional file 3) [24].

#### Comparing gene expression changes after a weight loss intervention between HR and LR

When the response to the intervention was compared between HR and LR at the pathway level, there was some overlap between HR and LR within studies (Additional file 4, Table 3). Adolescent males HR (Rendo-Urteaga et al.) had the most pathways enriched in response to the intervention (42 pathways) [24]. There were seventeen pathways commonly enriched between HR and LR to the intervention for Rendo-Urteaga et al. including the pathways ‘IL1 and megakaryocytes in obesity’, and ‘regulation of toll-like receptor signalling’ (Wikipathways: WP2865 and WP1449 respectively). HRs in Pinhel et al. had thirteen pathways enriched and HR in Harvie et al. had five pathways enriched in response to their respective interventions [22, 23]. There was no overlap in enriched pathways between HR and LR following the intervention in Harvie et al. [22].

**Fig. 5:**
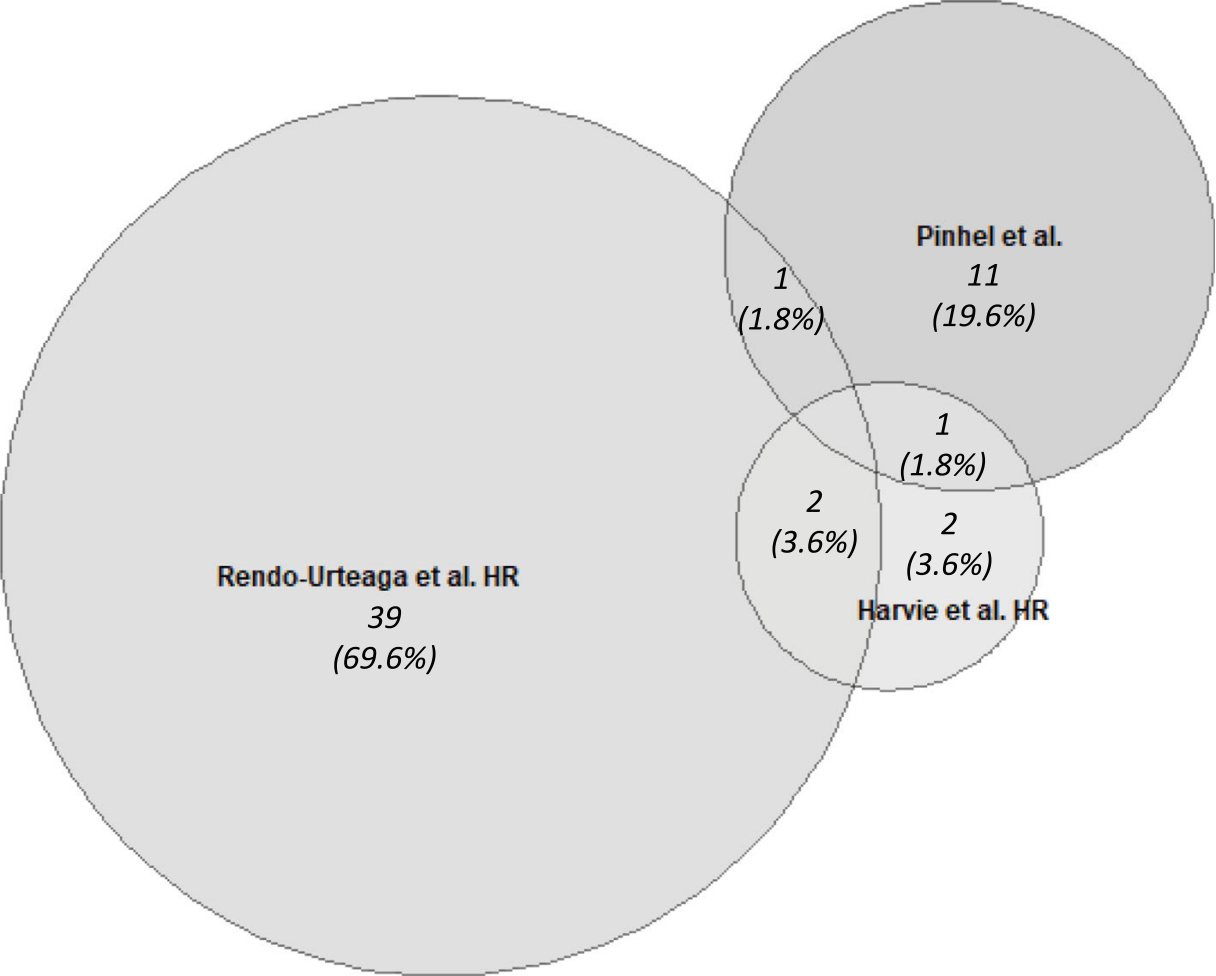
Euler diagram of pathways altered in HR in response to a weight loss intervention. Euler diagram created using eulerr package (version 6.1.0) in R (version 3.6.1) [29]

**Fig. 6:**
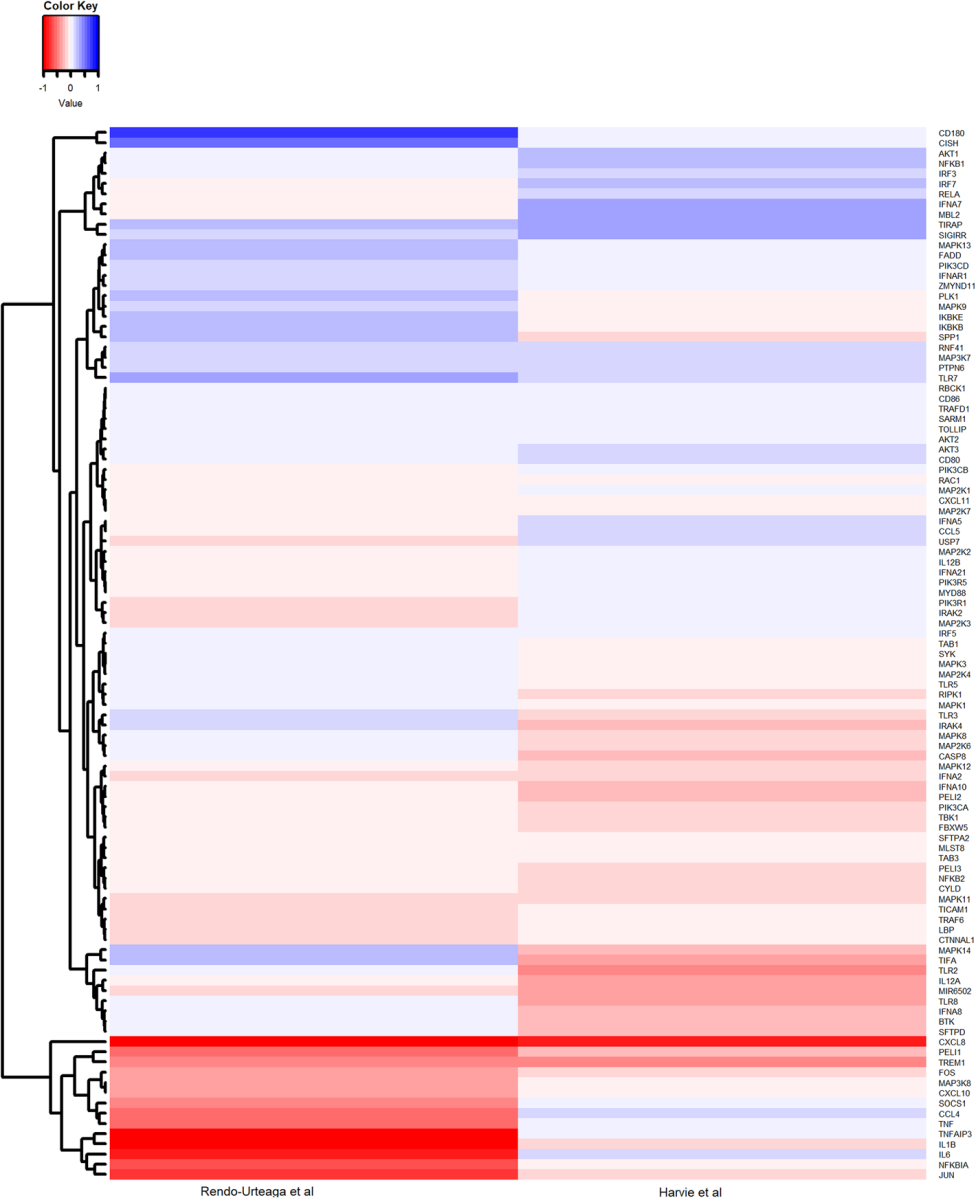
Heatmap and hierarchical clustering of genes involved in the two pathways relating to toll-like receptor signalling and regulation (WP1449 and WP75) that were enriched following the intervention in high responders in Rendo-Urteaga et al. and Harvie et al. Heatmaps were created using the gplots package (version 3.0.1.1.) in R (version 3.6.1). Columns represent studies and rows represent individual genes, where multiple probes mapped to one gene, expression of the probes was averaged to give one expression value per gene. Samples have been clustered using Euclidean distancing

When comparing the effect of the intervention on gene expression levels in HR between studies there was some overlap in enriched pathways **(**Fig. 5). There was one pathway enriched in HR in both Rendo-Urteaga et al. and Pinhel et al.: ‘circadian rhythm related genes’ (WikiPathways: WP3954) and one pathway enriched in HR in both Harvie et al. and Pinhel et al., ‘target of rapamycin (TOR) signalling’ (Wikipathways: WP1471) [22–24]. (Additional file 4). There were two pathways commonly enriched in HR between Rendo-Urteaga et al. and Harvie et al., ‘regulation of toll-like receptor signalling’ and ‘toll-like receptor signalling pathways’ (Wikipathways: WP1449 and WP75 respectively) [22, 24]. Specifically, with regard to HR in Harvie et al., there was a downregulation of genes involved in this pathway in particular *TLR1* *TLR4* and *TIRAP* (unadjusted *p* value < 0.05) [22]. Contrastingly, in HRs in Rendo-Urteaga et al., there was a downregulation of the cytokines activated by the pathways, specifically *TNF*, *IL1B*, *IL6* and *CCL3* and *CCL4* (unadjusted *p* value < 0.05) but a small, non-significant upregulation of TLR genes [24] **(**Fig. 6).

#### Discussion

This review aimed to describe transcriptomic changes that occur with weight loss and evaluate the similarities and differences between weight loss approaches. We report that transcriptomic changes at both the transcript and pathway level in PBMCs in response to weight loss interventions were small and highly variable, especially in adults. No transcripts were differentially expressed in all studies across all comparisons. However, seven pathways were impacted in two out of four studies in response to the weight loss interventions, and two pathways, ‘genes relating to cytoplasmic ribosomal proteins’, (Additional file 5) and ‘Initiation of transcription and translation elongation at the HIV-1 LTR’ in HR versus LR participants at baseline. Two pathways relating to toll-like receptor signalling were altered in HR in both Rendo-Urteaga et al. and Harvie et al. [22, 24].

The largest and most varied transcriptomic response to weight loss in PBMCs occurred in adolescent males, followed by adults who had bariatric surgery. Adolescents had modest weight loss after 10 weeks of calorie restriction (mean BMI SDS change: HR − 0.64, LR: − 0.07) compared with adults 6 months after bariatric surgery (mean weight loss: − 28.8 kg) [23, 24]. This suggests that a relatively smaller amount of weight loss is needed in adolescents to elicit a transcriptomic response in PBMCs compared to adults. It should be noted, however, that due to the lack of available phenotypic data, subgrouping of Pinhel et al. was not possible. Therefore, the differences in gene expression between Pinhel et al. and Rendo-Urteaga et al. could, in part, be explained by higher heterogeneity in response to bariatric surgery in adults; which may have hindered full investigation of the effect of the intervention in Pinhel et al. Rendo-Urteaga et al. is the only study to date to explore transcriptomic changes in PBMCs with weight loss in adolescents and whether this response is unique to this study design requires validation [24].

Stunted metabolic response to a stimulus such as weight loss demonstrates an inadequate ability to respond in a systematic and coordinated way [30]. Excess adiposity especially visceral adiposity drives a state of chronic low-grade inflammation linked to blunted metabolic adaptation [31]. There is evidence suggesting that adults and adolescents with obesity have different serum concentrations of particular cytokines which may, in part, explain the reduced responsiveness of the PBMC transcriptome to weight loss in adults in the studies reviewed here [32]. Indeed, it has been reported that there is a higher incidence of metabolically healthy obesity in adolescents than adults and that adolescents require a relatively smaller amount of weight loss which results in changes in circulating inflammatory markers such as adiponectin and CRP [33–35]. This suggests that intervention at an early age is important and that weight loss attempts in adults can be impacted by a host of complex factors that make weight loss challenging.

Differentially expressed genes and pathways were considered separately, using different criteria, as they provide different levels of information. Robust cut-offs are required to establish whether the transcription levels of a single gene can significantly change with weight loss or between responder groups whereas the clustering of genes on a given pathway is less likely to happen by chance and their cumulative change, whilst small in each instance, may collectively be biologically meaningful. The pathways ‘toll-like receptor signalling’ and ‘regulation of toll-like receptor (TLR) signalling’ (WP1449 and WP75 respectively) were enriched after the interventions in HR for Harvie et al. and Rendo-Urteaga et al. [22, 24]. TLR signalling activates proinflammatory cytokines and has been implicated in several tissues as mediators of obesity-induced proinflammation and insulin resistance in humans and mice [36–38]. Decreased activity of this signalling pathway, as seen in Rendo-Urteaga et al., suggests an attenuation of pro-inflammatory signalling in PBMCs with weight loss [24]. In contrast, this attenuation is not present in Harvie et al., and whilst over-represented, there was no clear directional change across the pathway, despite significant weight loss [22].

The pathway ‘cytoplasmic ribosomal proteins’ (WP477) was differentially expressed between HR and LR in both Rendo-Urteaga et al. and Harvie et al. with genes relating to this pathway generally decreased in HR in Harvie et al. and gene activity more varied in Rendo-Urteaga et al.’s HR [22, 24]. Ribosomes are responsible for protein synthesis. Analysis of gene expression in whole blood of participants with obesity has revealed upregulation of ribosomal proteins in obesity that may be due to increased metabolic demand [39]. Differential expression of this pathway could be due to differing energetic requirements.

Differences in intervention response both within and across studies highlight inconsistencies in gene expression responses to weight loss interventions. This raises the question of whether a HR to one intervention would necessarily be a HR in another. In order to be able to work towards utilising these data for therapeutic use, we need to work towards standardisation of biological material collection, reporting and data pooling. One limitation of pathway analysis is the presence of pathways with overlapping or similar functions allowing genes to be represented on multiple pathways which may lead to over-representation of genes of interest. Nevertheless, modulation of functionally similar pathways can indicate shifts in expression of broader biological functions.

The lack of commonality in response across studies may partially be explained by the high heterogeneity amongst participants, study designs and differences in the number of subjects in each subgroup. Whilst study design differences introduce variability, it allows for the exploration of whether transcriptomic changes in PBMCs in response to weight loss are conserved across a range of individuals and intervention designs, which is a strength of this review. It appears in the included studies that transcriptomic changes with weight loss are not consistently conserved. High heterogeneity amongst included studies has also allowed for the exploration of transcriptomic data within nutrition research in such a way that accommodates the inevitable variability within datasets and this approach could be applied with the inclusion of future studies. This is critical given the known high individual variability in response to dietary interventions. As was demonstrated, gene expression responses to the intervention were different between HR and LR, which may mask effects when assessing the transcriptomic response of the group as a whole.

Obesity itself is a complex and heterogenous condition with the potential for complications to arise in any tissue with only partially overlapping pathophysiology [40]. Small sample sizes often hinder the exploration of small, subtle changes in global gene expression, such as those observed with weight loss. To take full advantage of the depth of information available through transcriptomic analysis, standardised and detailed reporting is necessary, enabling the comparison of multiple studies, increasing sample sizes and the utility of these data. To minimise variability, there must be an increase in the consistency of reported data that are stored in an open-access and user-friendly format. Data sharing will support translation of research findings into practice, which remains elusive until this is realised. This is a reality which is rarely investigated but a widespread issue in nutrition research.

PBMCs are a heterogenous cell population which in itself introduces variability [41]. One of the limitations of this review is the inability to be able to quantify the sub-populations of cells within the included studies. Despite, or perhaps because of, the variability observed in the transcriptomic response to weight loss interventions, PBMCs remain a tissue of interest. PBMCs have previously shown good correlation with white adipose tissue in immune system genes but not with other processes, in particular lipid metabolism [42]. It is therefore not surprising that our results have yielded transcriptional modulation of genes involved in immune response, such as TLR signalling, but it is also important to note that these functions also have a role in obesity and its treatment. Many of the complications associated with obesity such as non-alcoholic fatty liver disease and type 2 diabetes are connected with inflammatory responses of tissues [5, 43, 44]. In order to make the best use of these data and to move towards a mechanistic understanding of weight loss, exploration of transcriptomic responses in conjunction with other tissue types is urgently needed.

Transcriptomic analysis shows promise in investigating phenotypic features that could be used to develop group-specific strategies. To achieve this, data reporting must be transparent and standardised. For example, a limitation in this review is that not all included transcriptomic datasets could be analysed alongside participant-level weight data. The use of multiple datasets is important as it has enabled the capture of variability and commonality across studies which cannot be seen when assessing single studies. Next, steps involve meta-analytic techniques that require raw gene expression data together with relevant phenotypic data, in order to address variability in transcriptomic responses. There is, therefore, a need for reporting standards for nutrigenomic studies that include detailed guidelines on reporting for collection, analysis and open-access availability of raw data and phenotypic outcomes. The recent OBEDIS guidelines take the first step towards standardisation of obesity research with the core variables required for weight loss interventions and is a stepping stone to work towards international standardisation within obesity research including omics technologies such as transcriptomics [45].

## Conclusions

In conclusion, this review shows that transcriptomic shifts in PBMCs do occur in response to weight loss. These shifts appear to be variable and, to date, present an inconsistent picture; however, variability itself may be a useful indicator of metabolic health and further exploration of this is needed. An integral part of moving this area of research forward lies in developing reporting standards that require transparency in method reporting and open access to transcriptomic and phenotypic data. Any move towards personalised weight management needs to be underpinned by a comprehensive understanding of the biological variation of obesity, and treatment response.

## Supplementary Information

**Additional file 1.** :Quality and Risk of Bias Assessment for included studies. Quality assessment tool and risk of bias assessment of included studies. If “Yes” were answered for eight or more questions studies were designated Positive, if eight or more answers were “No” the studies were designated Negative otherwise studies were designated Neutral

**Additional file 2:** Table of excluded studies. Table of studies excluded from the systematic review after full text screening with reasons for exclusion

**Additional file 3:** Significant genes lists for included studies. Tables of genes significantly differentially expressed in response to the intervention and between HR and LR at baseline for the studies which yielded significant genes.

**Additional file 4: **Pathway analysis tables. Pathways overrepresented in included studies when comparing differentially expressed genes (unadjusted p <0.05) in PBMCs between baseline and post-intervention and when comparing high and low responders at baseline (HR = reduction in body weight of ≥10%). **Table S1**. Pathways overrepresented when comparing differentially expressed genes (unadjusted p <0.05) between baseline and post intervention. **Table S2** Pathways overrepresented when comparing differentially expressed genes (unadjusted p <0.05) between high and low responders at baseline (HR = >10% body weight loss over the intervention period). **Table S3**. Pathways overrepresented in HR and LR responses to the intervention.

**Additional file 5: **PathVisio diagrams of the pathway “cytoplasmic ribosomal proteins” for the comparisons in which this pathway was significantly enriches. **Figure S1**. Harvie et al. Gene expression differences for the wikipathway ‘cytoplasmic ribosomal proteins’ in baseline compared to post-intervention. **Figure S2** Harvie et al. Gene expression differences for the wikipathway ‘cytoplasmic ribosomal proteins’ in high versus low responders at baseline. **Figure S3**. Rendo-Urteaga et al. Gene expression differences for the wikipathway ‘cytoplasmic ribosomal proteins’ in high versus low responders at baseline.

**Additional file 6:** PRISMA checklist for Systematic Literature Reviews. Description of data: PRISMA checklist for standardised reporting and minimum requirements for systematic literature reviews.

## Data Availability

Data described in the manuscript is publicly and freely available at: https://www.ncbi.nlm.nih.gov/geo/ under accession numbers: GSE66161, GSE83223, GSE41505 and GSE88794.

## Abbreviations

PBMCs: Peripheral blood mononuclear cells
HR: High responder
LR: Low responder
RMA: Robust multi-array averaging
TLR: Toll-like receptor
