# Supplementary material for: Transcriptomic changes in peripheral blood mononuclear cells with weight loss: systematic literature review and primary data synthesis
Source: Genes Nutr. 2021 Jul 19;16:12. doi: 10.1186/s12263-021-00692-6 (PMC8287703; doi:10.1186/s12263-021-00692-6)
Supplement: Supplementary file 1 — Additional file 1. :Quality and Risk of Bias Assessment for included studies. Quality assessment tool and risk of bias assessment of included studies. If “Yes” were answered for eight or more questions studies were designated Positive, if eight or more answers were “No” the studies were designated Negative otherwise studies were designated Neutral [file 12263_2021_692_MOESM1_ESM.docx]

| ***Study (1st Author, Year)*** | **Crujeiras et al** | **Harvie et al** | **Pinhel et al 2017 and 2018** | **Rendo-Urteaga et al** | **Samblas et al** | **vanBussel et al** |
| --- | --- | --- | --- | --- | --- | --- |
| *Q1* | Y | N | Y | Y | Y | Y |
| *Q2* | Y | Y | Y | Y | Y | Y |
| *Q3* | N | Y | N | Y | Y | Y |
| *Q4* | NA | NA | Y | NA | NA | Y |
| *Q5* | NA | NA | N | NA | NA | NA |
| *Q6* | N | Y | Y | Y | Y | Y |
| *Q7* | N | Y | Y | Y | Y | Y |
| *Q8* | N | N | N | N | N | N |
| *Q9* | NA | NA | NA | NA | NA | NA |
| *Q10* | N | Y | Y | Y | Y | Y |
| *Q11* | N | Y | Y | Y | Y | Y |
| *Q12* | N | N | N | Y | N | Y |
| *Q13* | N | Y | N | Y | Y | Y |
| *Q14* | N | N | N | N | N | N |
| *Q15* | Y | Y | Y | Y | Y | Y |
| **Overall** | NEG | P | NEU | P | NEU | P |

Additional file 1. Quality and Risk of Bias Assessment for included studies. If “Yes” were answered for 8 or more questions studies were designated Positive, if 8 or more answers were “No” the studies were designated Negative otherwise studies were designated Neutral. Adapted from (1).

The following questions were used for quality assessment and risk of bias:

| **VALIDITY QUESTIONS - PRIMARY STUDIES** |
| --- |
| *1. Was the research question clearly stated?* |
| *2. Was the study design clearly stated?* |
| *3. Was the selection of study subjects/patients free from bias? Was the eligibility criteria clearly stated along with the sources and methods of selection of participants/cases* |
| *4. Were study groups comparable?* |
| *5. If studies were matched was the matching criteria clearly outlined?* |
| *6. Were intervention/therapeutic regimens/exposure factor or procedure and any comparison(s) described in detail? Were intervening factors described?* |
| *7. Were outcomes clearly defined and the measurements valid and reliable?* |
| *8. Was method of handling withdrawals described?* |
| *9. Was blinding used to prevent introduction of bias?* |
| *10. Were statistical methods appropriately described?* |
| *11. Was the statistical analysis appropriate for the study design and type of outcome indicators?* |
| *12. Were appropriate quality control checks performed on expression data and clearly reported?* |
| *13. Were conclusions supported by results with biases and limitations taken into consideration?* |
| *14. Was the direction and magnitude of any potential biases discussed?* |
| *15. Is bias due to study’s funding or sponsorship unlikely?* |
|  |
| **Negative/Neutral/Positive (N/0/P)** |
| **If most (eight or more) of the answers to the above validity questions are “No,” the report should be designated negative** |
| **If the answers to validity criteria questions 3, 4, 6, and 7 do not indicate that the study is exceptionally strong, the report should be designated neutral** |
| **If most of the answers to the above validity questions are “Yes” (including criteria 2, 3, 6, 7 and at least one additional “Yes”), the report should be designated positive** |

References:

1. Day KJ, Adamski MM, Dordevic AL, Murgia C. Genetic variations as modifying factors to dietary zinc requirements—A systematic review. Nutrients. 2017;9(2):148.
