# Supplementary material for: Transcriptomic changes in peripheral blood mononuclear cells with weight loss: systematic literature review and primary data synthesis
Source: Genes Nutr. 2021 Jul 19;16:12. doi: 10.1186/s12263-021-00692-6 (PMC8287703; doi:10.1186/s12263-021-00692-6)
Supplement: Supplementary file 2 — Additional file 2: Table of excluded studies. Table of studies excluded from the systematic review after full text screening with reasons for exclusion [file 12263_2021_692_MOESM2_ESM.docx]

Additional file 2. Table summarising all articles excluded in the second pass screening with reasons (n=41).

| First Author | Year Published | Title | Exclusion Reason |
| --- | --- | --- | --- |
| Bakker, G | 2010 | An anti-inflammatory dietary mix modulates inflammation and oxidative and metabolic stress in overweight men: a nutrigenomics approach | Not an intervention of interest |
| Berisha, Stela Z | 2011 | Changes in whole blood gene expression in obese subjects with type 2 diabetes following bariatric surgery: a pilot study | Not a tissue type of interest |
| Brattbakk, H | 2013 | Balanced caloric macronutrient composition downregulates immunological gene expression in human blood cells - Adipose tissue diverges | Not a tissue type of interest |
| Bye, A | 2009 | Transcriptional changes in blood after aerobic interval training in patients with the metabolic syndrome | Not a tissue type of interest |
| Camargo, A | 2010 | Gene expression changes in mononuclear cells in patients with metabolic syndrome after acute intake of phenol-rich virgin olive oil | Not an intervention of interest |
| Chachay, V. S | 2014 | Resveratrol Does Not Benefit Patients With Nonalcoholic Fatty Liver Disease | Not an intervention of interest |
| Chacko, S. A | 2011 | Magnesium supplementation, metabolic and inflammatory markers, and global genomic and proteomic profiling: A randomized, double-blind, controlled, crossover trial in overweight individuals | Not an intervention of interest |
| Cruz-Teno, C | 2012 | Dietary fat modifies the postprandial inflammatory state in subjects with metabolic syndrome: The LIPGENE study | Not an intervention of interest |
| Edwards, C | 2011 | Downregulation of leptin and resistin expression in blood following bariatric surgery | Not a tissue type of interest |
| Ellsworth, D. L | 2015 | Importance of substantial weight loss for altering gene expression during cardiovascular lifestyle modification | Not a tissue type of interest |
| Garcia-Lacarte, M | 2018 | Implication of miR-612 and miR-1976 in the regulation of TP53 and CD40 and their relationship in the response to specific weight-loss diets | Not a tissue type of interest |
| Garcia-Ruiz, E | 2015 | The intake of high-fat diets induces the acquisition of brown adipocyte gene expression features in white adipose tissue | Not a population of interest |
| Ghanim, H. | 2018 | Decreases in neprilysin and vasoconstrictors and increases in vasodilators following bariatric surgery | Global gene expression not measured |
| Ghanim, H | 2012 | Reduction in inflammation and the expression of amyloid precursor protein and other proteins related to Alzheimer's disease following gastric bypass surgery | Global gene expression not measured |
| Ghosh, S | 2011 | Blood gene expression reveal pathway differences between diet-sensitive and resistant obese subjects prior to caloric restriction | Not a tissue type of interest |
| Gruchala-Niedoszytko, M | 2018 | Differences in gene expression related to the outcomes of obesity treatment, peak oxygen uptake, and fatty acid metabolism measured in a cardiopulmonary exercise test | Not a tissue type of interest |
| Heilbronn, L. K | 2007 | Markers of mitochondrial biogenesis and metabolism are lower in overweight and obese insulin-resistant subjects | Not a tissue type of interest |
| Henagan, T. M. | 2011 | The Melanocortin 3 receptor: A novel mediator of exercise-induced inflammation reduction in postmenopausal women? | Global gene expression not measured |
| Hindle, A | 2010 | Reactivation of adiponectin expression in obese patients after bariatric surgery | Not a tissue type of interest |
| Hofer, T | 2008 | Long-term effects of caloric restriction or exercise on DNA and RNA oxidation levels in white blood cells and urine in humans | Not an outcome of interest |
| Hulsmans, M | 2012 | Interleukin-1 receptor-associated kinase-3 is a key inhibitor of inflammation in obesity and metabolic syndrome | Not a tissue type of interest |
| Kolehmainen, M | 2012 | Bilberries reduce low-grade inflammation in individuals with features of metabolic syndrome | Not an intervention of interest |
| Lopez-Moreno, J | 2017 | Effect of Dietary Lipids on Endotoxemia Influences Postprandial Inflammatory Response | Global gene expression not measured |
| Miranda, D | 2014 | Increases in insulin sensitivity among obese youth are associated with gene expression changes in whole blood | Not a tissue type of interest |
| Obata, Y | 2016 | Impact of visceral fat on gene expression profile in peripheral blood cells in obese Japanese subjects | Not a tissue type of interest |
| Oh, S | 2017 | High-Intensity Aerobic Exercise Improves Both Hepatic Fat Content and Stiffness in Sedentary Obese Men with Nonalcoholic Fatty Liver Disease | Global gene expression not measured |
| Pardina, E | 2016 | Diabetic and dyslipidaemic morbidly obese exhibit more liver alterations compared with healthy morbidly obese | Not a tissue type of interest |
| Pardina, E | 2017 | Hepatic CD36 downregulation parallels steatosis improvement in morbidly obese undergoing bariatric surgery | Not a tissue type of interest |
| Pei, R | 2017 | Low-fat yogurt consumption reduces biomarkers of chronic inflammation and inhibits markers of endotoxin exposure in healthy premenopausal women: a randomised controlled trial | Not an intervention of interest |
| Radler U | 2011 | A combination of (-3) polyunsaturated fatty acids, polyphenols and L-carnitine reduces the plasma lipid levels and increases the expression of genes involved in fatty acid oxidation in human peripheral blood mononuclear cells and HepG2 cells | Not an intervention of interest |
| Rainone, V | 2016 | Upregulation of inflammasome activity and increased gut permeability are associated with obesity in children and adolescents | Global gene expression not measured |
| Rampersaud, E | 2013 | Genomic signatures of a global fitness index in a multi-ethnic cohort of women | Not a tissue type of interest |
| Rosqvist, F | 2015 | Potential role of milk fat globule membrane in modulating plasma lipoproteins, gene expression, and cholesterol metabolism in humans: a randomized study | Not an intervention of interest |
| Rudkowska, I | 2011 | Effects of a supplementation of n-3 polyunsaturated fatty acids with or without fish gelatin on gene expression in peripheral blood mononuclear cells in obese, insulin-resistant subjects | Not an intervention of interest |
| Rudkowska, I | 20111 | Validation of the use of peripheral blood mononuclear cells as surrogate model for skeletal muscle tissue in nutrigenomic studies | Not an intervention of interest |
| Trachta, P | 2014 | Laparoscopic sleeve gastrectomy ameliorates mRNA expression of inflammation-related genes in subcutaneous adipose tissue but not in peripheral monocytes of obese patients | Global gene expression not measured |
| van der Velpen, V | 2016 | A risk assessment-driven quantitative comparison of gene expression profiles in PBMCs and white adipose tissue of humans and rats after isoflavone supplementation | Not an intervention of interest |
| van Dijk, S. J | 2012 | Consumption of a high monounsaturated fat diet reduces oxidative phosphorylation gene expression in peripheral blood mononuclear cells of abdominally overweight men and women | Not an intervention of interest |
| Wang, J. | 2011 | Soy food supplementation, dietary fat reduction and peripheral blood gene expression in postmenopausal women - A randomized, controlled trial | Not an intervention of interest |
