# Supplementary material for: Transcriptomic changes in peripheral blood mononuclear cells with weight loss: systematic literature review and primary data synthesis
Source: Genes Nutr. 2021 Jul 19;16:12. doi: 10.1186/s12263-021-00692-6 (PMC8287703; doi:10.1186/s12263-021-00692-6)
Supplement: Supplementary file 4 — Additional file 4: Pathway analysis tables. Pathways overrepresented in included studies when comparing differentially expressed genes (unadjusted p <0.05) in PBMCs between baseline and post-intervention and when comparing high and low responders at baseline (HR = reduction in body weight of ≥10%). Table S1. Pathways overrepresented when comparing differentially expressed genes (unadjusted p <0.05) between baseline and post intervention. Table S2 Pathways overrepresented when comparing differentially expressed genes (unadjusted p <0.05) between high and low responders at baseline (HR = >10% body weight loss over the intervention period). Table S3. Pathways overrepresented in HR and LR responses to the intervention. [file 12263_2021_692_MOESM4_ESM.docx]

Additional file 4. Tables 1, 2 and 3.

Pathways overrepresented in included studies when comparing differentially expressed genes (unadjusted p <0.05) in PBMCs between baseline and post-intervention and when comparing high and low responders at baseline (HR = reduction in body weight of ≥10%).

Table 1. Pathways overrepresented when comparing differentially expressed genes (unadjusted p <0.05) between baseline and post intervention.

| Pathway | Number of positive genes | Percentage of total genes | Z-score |
| --- | --- | --- | --- |
| Harvie et al | | | |
| Allograft Rejection | 13 | 14.94% | 4.97 |
| Cytoplasmic ribosomal proteins | 6 | 12.00% | 2.73 |
| TYROBP Causal Network | 6 | 10.53% | 2.36 |
| Lung fibrosis | 6 | 10.53% | 2.22 |
| Endochondral Ossification | 6 | 9.84% | 2.17 |
| Spinal Cord Injury | 9 | 8.26% | 2.09 |
| AGE/RAGE Pathway | 6 | 9.52% | 2.08 |
| Viral Acute Myocarditis | 7 | 8.86% | 2.04 |
| Rendo-Urteaga et al | | | |
| Apoptosis Modulation and Signaling | 34 | 40.96% | 4.51 |
| Exercise-induced Circadian Regulation | 20 | 48.78% | 4.4 |
| Apoptosis | 31 | 37.80% | 3.78 |
| IL-1 signaling pathway | 20 | 41.67% | 3.55 |
| Nucleotide-binding Oligomerization Domain (NOD) pathway | 17 | 43.59% | 3.49 |
| Apoptosis Modulation by HSP70 | 9 | 56.25% | 3.48 |
| Regulation of toll-like receptor signaling pathway | 42 | 32.81% | 3.34 |
| IL-4 Signaling Pathway | 19 | 40.43% | 3.3 |
| Toll-like Receptor Signaling Pathway | 31 | 34.83% | 3.25 |
| DNA Replication | 16 | 42.11% | 3.22 |
| Mammary gland development pathway - Puberty (Stage 2 of 4) | 7 | 58.33% | 3.19 |
| Chromosomal and microsatellite instability in colorectal cancer | 24 | 36.92% | 3.19 |
| TNF related weak inducer of apoptosis (TWEAK) Signaling Pathway | 16 | 41.03% | 3.1 |
| Photodynamic therapy-induced NF-kB survival signaling | 14 | 42.42% | 3.04 |
| TP53 Network | 9 | 50.00% | 3.04 |
| Copper homeostasis | 19 | 38.00% | 2.98 |
| B Cell Receptor Signaling Pathway | 30 | 33.33% | 2.91 |
| Canonical NF-KB pathway | 5 | 62.50% | 2.89 |
| Signal transduction through IL1R | 13 | 41.94% | 2.88 |
| TGF-beta Signaling Pathway | 37 | 31.62% | 2.87 |
| miRNAs involvement in the immune response in sepsis | 20 | 36.36% | 2.83 |
| Translation Factors | 17 | 37.78% | 2.79 |
| Structural Pathway of Interleukin 1 (IL-1) | 16 | 38.10% | 2.75 |
| Transcriptional cascade regulating adipogenesis | 6 | 54.55% | 2.74 |
| mRNA Processing | 35 | 31.25% | 2.71 |
| Oncostatin M Signaling Pathway | 19 | 35.85% | 2.68 |
| Glycosylation and related congenital defects | 10 | 43.48% | 2.66 |
| Apoptosis-related network due to altered Notch3 in ovarian cancer | 16 | 37.21% | 2.63 |
| miRNAs involved in DNA damage response | 17 | 36.17% | 2.58 |
| Role Altered Glycolysation of MUC1 in Tumour Microenvironment | 5 | 55.56% | 2.56 |
| TGF-beta Receptor Signaling | 18 | 35.29% | 2.53 |
| RANKL/RANK (Receptor activator of NFKB (ligand)) Signaling Pathway | 17 | 35.42% | 2.48 |
| Vitamin D in inflammatory diseases | 9 | 42.86% | 2.48 |
| p38 MAPK Signaling Pathway | 12 | 38.71% | 2.44 |
| MAPK Signaling Pathway | 61 | 27.48% | 2.44 |
| RIG-I-like Receptor Signaling | 19 | 33.93% | 2.4 |
| TNF alpha Signaling Pathway | 27 | 31.40% | 2.4 |
| Interferon type I signaling pathways | 17 | 34.69% | 2.38 |
| miRNA Regulation of DNA Damage Response | 25 | 31.65% | 2.36 |
| MicroRNAs in cardiomyocyte hypertrophy | 28 | 30.77% | 2.32 |
| Thymic Stromal LymphoPoietin (TSLP) Signaling Pathway | 14 | 35.90% | 2.31 |
| Fas Ligand (FasL) pathway and Stress induction of Heat Shock Proteins (HSP) regulation | 14 | 35.90% | 2.31 |
| Non-genomic actions of 1,25 dihydroxyvitamin D3 | 20 | 32.79% | 2.29 |
| G1 to S cell cycle control | 20 | 32.79% | 2.29 |
| Ciliary landscape | 53 | 27.46% | 2.26 |
| T-Cell antigen Receptor (TCR) Signaling Pathway | 25 | 30.86% | 2.21 |
| Pancreatic adenocarcinoma pathway | 24 | 30.77% | 2.15 |
| Circadian rhythm related genes | 49 | 27.37% | 2.15 |
| Cardiac Hypertrophic Response | 16 | 33.33% | 2.12 |
| Development and heterogeneity of the ILC family | 10 | 37.04% | 2.06 |
| Integrated Breast Cancer Pathway | 43 | 27.56% | 2.06 |
| Simplified Depiction of MYD88 Distinct Input-Output Pathway | 7 | 41.18% | 2.06 |
| MAPK and NFkB Signalling Pathways Inhibited by Yersinia YopJ | 5 | 45.45% | 2 |
| Photodynamic therapy-induced unfolded protein response | 9 | 37.50% | 2 |
| Pinhel et al | | | |
| Lamin A-processing pathway | 5 | 100.00% | 4.78 |
| Common Pathways Underlying Drug Addiction | 8 | 44.44% | 2.93 |
| Target Of Rapamycin (TOR) Signaling | 10 | 35.71% | 2.45 |
| Heart Development | 6 | 42.86% | 2.43 |
| Urea cycle and associated pathways | 5 | 45.45% | 2.37 |
| miRNAs involved in DNA damage response | 6 | 40.00% | 2.22 |
| Circadian rhythm related genes | 27 | 26.21% | 2.2 |
| Vitamin A and Carotenoid Metabolism | 5 | 41.67% | 2.14 |
| Sterol Regulatory Element-Binding Proteins (SREBP) signalling | 14 | 29.79% | 2.12 |
| Wnt Signaling Pathway and Pluripotency | 14 | 29.17% | 2.03 |
| Association Between Physico-Chemical Features and Toxicity Associated Pathways | 12 | 30.00% | 1.99 |
| Hair Follicle Development: Cytodifferentiation (Part 3 of 3) | 8 | 33.33% | 1.96 |
| vanBussel et al |  |  |  |
| TYROBP Causal Network | 27 | 49.09% | 6.47 |
| Microglia Pathogen Phagocytosis Pathway | 16 | 42.11% | 4.22 |
| Interleukin-11 Signaling Pathway | 15 | 34.88% | 3.21 |
| Metabolic reprogramming in colon cancer | 13 | 32.50% | 2.69 |
| B Cell Receptor Signaling Pathway | 24 | 26.97% | 2.62 |
| Apoptosis Modulation and Signaling | 21 | 26.92% | 2.44 |
| Viral Acute Myocarditis | 20 | 26.67% | 2.34 |
| Fatty Acid Beta Oxidation | 10 | 32.26% | 2.33 |
| Fibrin Complement Receptor 3 Signaling Pathway | 10 | 32.26% | 2.33 |
| IL-3 Signaling Pathway | 13 | 29.55% | 2.3 |
| Amyotrophic lateral sclerosis (ALS) | 11 | 30.56% | 2.24 |
| Alpha 6 Beta 4 signaling pathway | 10 | 31.25% | 2.22 |
| Sphingolipid pathway | 7 | 33.33% | 2.05 |
| Integrin-mediated Cell Adhesion | 22 | 24.72% | 2.05 |
| IL-4 Signaling Pathway | 14 | 26.92% | 1.99 |

Table 2. Pathways overrepresented when comparing differentially expressed genes (unadjusted p <0.05) between high and low responders at baseline (HR = >10% body weight loss over the intervention period).

| Pathway | Number of positive genes | Percentage of total genes | Z-score |
| --- | --- | --- | --- |
| Rendo-Urteaga et al |  | | |
| Lamin A-processing pathway | 5 | 100.00% | 6.46 |
| Supression of HMGB1 mediated inflammation by THBD | 5 | 55.56% | 4.35 |
| Endothelin Pathways | 10 | 31.25% | 3.77 |
| Osteopontin Signaling | 5 | 38.46% | 3.24 |
| Synaptic Vesicle Pathway | 12 | 23.53% | 2.97 |
| Overview of nanoparticle effects | 6 | 31.58% | 2.95 |
| NRF2 pathway | 25 | 18.38% | 2.92 |
| miRNAs involvement in the immune response in sepsis | 13 | 21.31% | 2.69 |
| TGF-beta Receptor Signaling | 12 | 21.82% | 2.67 |
| AGE/RAGE pathway | 13 | 20.00% | 2.43 |
| miRNA targets in ECM and membrane receptors | 9 | 22.50% | 2.42 |
| Eicosanoid Synthesis | 6 | 26.09% | 2.39 |
| ncRNAs involved in STAT3 signaling in hepatocellular carcinoma | 5 | 27.78% | 2.34 |
| Cytoplasmic Ribosomal Proteins | 16 | 18.39% | 2.33 |
| Selenium Micronutrient Network | 15 | 18.29% | 2.23 |
| Glutathione metabolism | 5 | 26.32% | 2.2 |
| Hypothesized Pathways in Pathogenesis of Cardiovascular Disease | 6 | 24.00% | 2.15 |
| VEGFA-VEGFR2 Signaling Pathway | 35 | 14.83% | 2.08 |
| Initiation of transcription and translation elongation at the HIV-1 LTR | 7 | 21.88% | 2.05 |
| Myometrial Relaxation and Contraction Pathways | 24 | 15.58% | 1.97 |
| Resistin as a regulator of inflammation | 7 | 21.21% | 1.96 |
| Harvie et al |  | | |
| Cancer immunotherapy by PD-1 blockade | 5 | 29.41% | 3.94 |
| Exercise-induced Circadian Regulation | 9 | 20.00% | 3.80 |
| 4-hydroxytamoxifen, Dexamethasone, and Retinoic Acids Regulation of p27 Expression | 5 | 27.78% | 3.76 |
| Translation inhibitors in chronically activated PDGFRA cells | 8 | 19.51% | 3.50 |
| Ferroptosis | 7 | 19.44% | 3.26 |
|  |  |  |  |
| Structural Pathway of Interleukin 1 (IL-1) | 8 | 16.67% | 2.98 |
| Cytoplasmic Ribosomal Proteins | 8 | 16.00% | 2.84 |
| Regulation of Microtubule Cytoskeleton | 7 | 15.56% | 2.57 |
| T-Cell Receptor and Co-stimulatory Signaling | 5 | 17.86% | 2.53 |
| DNA Damage Response (only ATM dependent) | 13 | 11.82% | 2.41 |
| Ebola Virus Pathway on Host | 14 | 11.38% | 2.35 |
| Initiation of transcription and translation elongation at the HIV-1 LTR | 5 | 16.67% | 2.35 |
| Glycogen Synthesis and Degradation | 6 | 14.63% | 2.21 |
| mRNA Processing | 13 | 11.21% | 2.20 |
|  |  |  |  |
| Wnt Signaling Pathway and Pluripotency | 11 | 11.70% | 2.18 |
| Nonalcoholic fatty liver disease | 14 | 10.85% | 2.16 |
| Retinoblastoma Gene in Cancer | 10 | 11.90% | 2.14 |
| Target Of Rapamycin (TOR) Signaling | 5 | 15.15% | 2.11 |
| Insulin Signaling | 16 | 10.13% | 2.01 |
| Ciliary landscape | 19 | 9.69% | 1.99 |

Table 3. Pathways overrepresented in HR and LR responses to the intervention. (HR = >10% body weight loss over the intervention period).

| Pathway | Number of positive genes | Percentage of total genes | Z-score |
| --- | --- | --- | --- |
| Rendo-Urteaga et al HR |  | | |
| TGF-beta Receptor Signaling | 16 | 32.00% | 4.14 |
| Lung fibrosis | 18 | 29.51% | 3.99 |
| Cytoplasmic Ribosomal Proteins | 23 | 26.74% | 3.97 |
| Mammary gland development pathway - Puberty (Stage 2 of 4) | 6 | 50.00% | 3.91 |
| Vitamin D in inflammatory diseases | 8 | 40.00% | 3.7 |
| Photodynamic therapy-induced NF-kB survival signaling | 10 | 31.25% | 3.18 |
| Circadian rhythm related genes | 36 | 20.45% | 3.17 |
| IL-1 signaling pathway | 13 | 27.66% | 3.12 |
| NOTCH1 regulation of human endothelial cell calcification | 6 | 37.50% | 3 |
| Apoptosis Modulation and Signaling | 19 | 23.46% | 2.96 |
| TNF related weak inducer of apoptosis (TWEAK) Signaling Pathway | 11 | 28.21% | 2.94 |
| Apoptosis | 18 | 23.38% | 2.86 |
| RANKL/RANK (Receptor activator of NFKB (ligand)) Signaling Pathway | 12 | 26.09% | 2.76 |
| Development and heterogeneity of the ILC family | 8 | 29.63% | 2.67 |
| Small Ligand GPCRs | 6 | 33.33% | 2.65 |
| Zinc homeostasis | 9 | 28.13% | 2.65 |
| Fas Ligand (FasL) pathway and Stress induction of Heat Shock Proteins (HSP) regulation | 10 | 27.03% | 2.65 |
| Hypothesized Pathways in Pathogenesis of Cardiovascular Disease | 7 | 30.43% | 2.58 |
| Thymic Stromal LymphoPoietin (TSLP) Signaling Pathway | 11 | 25.58% | 2.57 |
| Signal transduction through IL1R | 8 | 28.57% | 2.55 |
| B Cell Receptor Signaling Pathway | 19 | 21.11% | 2.44 |
| Regulation of toll-like receptor signaling pathway | 24 | 19.67% | 2.37 |
| Selective expression of chemokine receptors during T-cell polarization | 7 | 28.00% | 2.32 |
| Chromosomal and microsatellite instability in colorectal cancer | 15 | 21.74% | 2.3 |
| Oncostatin M Signaling Pathway | 13 | 22.41% | 2.26 |
| Canonical and Non-Canonical TGF-B signaling | 5 | 31.25% | 2.25 |
| Differentiation Pathway | 9 | 25.00% | 2.25 |
| MicroRNAs in cardiomyocyte hypertrophy | 18 | 20.45% | 2.23 |
| TGF-beta Signaling Pathway | 23 | 19.33% | 2.23 |
| Toll-like Receptor Signaling Pathway | 17 | 20.48% | 2.17 |
| Structural Pathway of Interleukin 1 (IL-1) | 10 | 23.26% | 2.11 |
| TGF-B Signaling in Thyroid Cells for Epithelial-Mesenchymal Transition | 5 | 29.41% | 2.09 |
| Ethanol effects on histone modifications | 7 | 25.93% | 2.09 |
| IL-4 Signaling Pathway | 11 | 22.45% | 2.08 |
| IL1 and megakaryocytes in obesity | 6 | 27.27% | 2.08 |
| Genotoxicity pathway | 12 | 21.82% | 2.07 |
| miRNAs involvement in the immune response in sepsis | 12 | 21.82% | 2.07 |
| Exercise-induced Circadian Regulation | 10 | 22.73% | 2.03 |
| Neural Crest Cell Migration during Development | 8 | 24.24% | 2.02 |
| One Carbon Metabolism | 7 | 25.00% | 1.98 |
| p38 MAPK Signaling Pathway | 7 | 25.00% | 1.98 |
| DNA Replication | 9 | 23.08% | 1.98 |
| Rendo-Urteaga et al LR | | | |
| Regulation of toll-like receptor signaling pathway | 30 | 21.90% | 3.87 |
| Apoptosis Modulation and Signaling | 21 | 24.14% | 3.73 |
| IL-1 signaling pathway | 14 | 27.45% | 3.6 |
| RIG-I-like Receptor Signaling | 15 | 26.32% | 3.53 |
| Apoptosis | 19 | 23.46% | 3.41 |
| Nucleotide-binding Oligomerization Domain (NOD) pathway | 10 | 28.57% | 3.18 |
| Exercise-induced Circadian Regulation | 12 | 26.09% | 3.12 |
| Apoptosis Modulation by HSP70 | 6 | 35.29% | 3.09 |
| Simplified Depiction of MYD88 Distinct Input-Output Pathway | 6 | 35.29% | 3.09 |
| Hypertrophy Model | 6 | 35.29% | 3.09 |
| miRNAs involvement in the immune response in sepsis | 14 | 24.14% | 3.04 |
| miRNAs involved in DNA damage response | 12 | 25.53% | 3.04 |
| IL1 and megakaryocytes in obesity | 7 | 31.82% | 3 |
| Thymic Stromal LymphoPoietin (TSLP) Signaling Pathway | 11 | 25.58% | 2.91 |
| Aryl Hydrocarbon Receptor Pathway | 9 | 27.27% | 2.86 |
| Toll-like Receptor Signaling | 8 | 28.57% | 2.85 |
| mRNA Processing | 22 | 19.64% | 2.74 |
| B Cell Receptor Signaling Pathway | 19 | 20.43% | 2.73 |
| IL-4 Signaling Pathway | 12 | 23.08% | 2.64 |
| Toll-like Receptor Signaling Pathway | 19 | 19.79% | 2.58 |
| Chromosomal and microsatellite instability in colorectal cancer | 14 | 21.54% | 2.56 |
| Signaling of Hepatocyte Growth Factor Receptor | 8 | 25.81% | 2.51 |
| Mammary gland development pathway - Embryonic development (Stage 1 of 4) | 5 | 31.25% | 2.49 |
| TGF-beta Signaling Pathway | 22 | 18.64% | 2.47 |
| miRNA regulation of p53 pathway in prostate cancer | 6 | 28.57% | 2.46 |
| Signal transduction through IL1R | 8 | 25.00% | 2.41 |
| Structural Pathway of Interleukin 1 (IL-1) | 10 | 22.73% | 2.35 |
| Interferon type I signaling pathways | 11 | 21.57% | 2.27 |
| Photodynamic therapy-induced unfolded protein response | 6 | 26.09% | 2.21 |
| Interactions between immune cells and microRNAs in tumor microenvironment | 10 | 21.74% | 2.19 |
| Resistin as a regulator of inflammation | 7 | 24.14% | 2.15 |
| TGF-beta Receptor Signaling | 11 | 20.75% | 2.13 |
| Metastatic brain tumor | 6 | 25.00% | 2.09 |
| Photodynamic therapy-induced NF-kB survival signaling | 7 | 23.33% | 2.05 |
| Selenium Metabolism and Selenoproteins | 8 | 22.22% | 2.03 |
| Hepatitis C and Hepatocellular Carcinoma | 10 | 20.41% | 1.97 |
| Factors and pathways affecting insulin-like growth factor (IGF1)-Akt signaling | 6 | 24.00% | 1.97 |
| Harvie et al HR | | | |
| Target Of Rapamycin (TOR) Signaling | 5 | 15.15% | 4.88 |
| Pathways in clear cell renal cell carcinoma | 6 | 7.59% | 3.11 |
| Regulation of toll-like receptor signaling pathway | 7 | 5.47% | 2.36 |
| PI3K-Akt Signaling Pathway | 13 | 4.14% | 2.16 |
| Toll-like Receptor Signaling Pathway | 5 | 5.49% | 2 |
| Harvie et al LR | | | |
| Type II interferon signaling (IFNG) | 7 | 21.21% | 7.04 |
| Non-genomic actions of 1,25 dihydroxyvitamin D3 | 5 | 7.14% | 2.58 |
| Retinoblastoma Gene in Cancer | 5 | 5.95% | 2.12 |
| Spinal Cord Injury | 6 | 5.50% | 2.11 |
