# Supplementary material for: Transcriptomic changes in peripheral blood mononuclear cells with weight loss: systematic literature review and primary data synthesis
Source: Genes Nutr. 2021 Jul 19;16:12. doi: 10.1186/s12263-021-00692-6 (PMC8287703; doi:10.1186/s12263-021-00692-6)

Additional file 5

Figure 1. Harvie et al. Gene expression differences for the wikipathway ‘cytoplasmic ribosomal proteins’ in baseline compared to post-intervention.


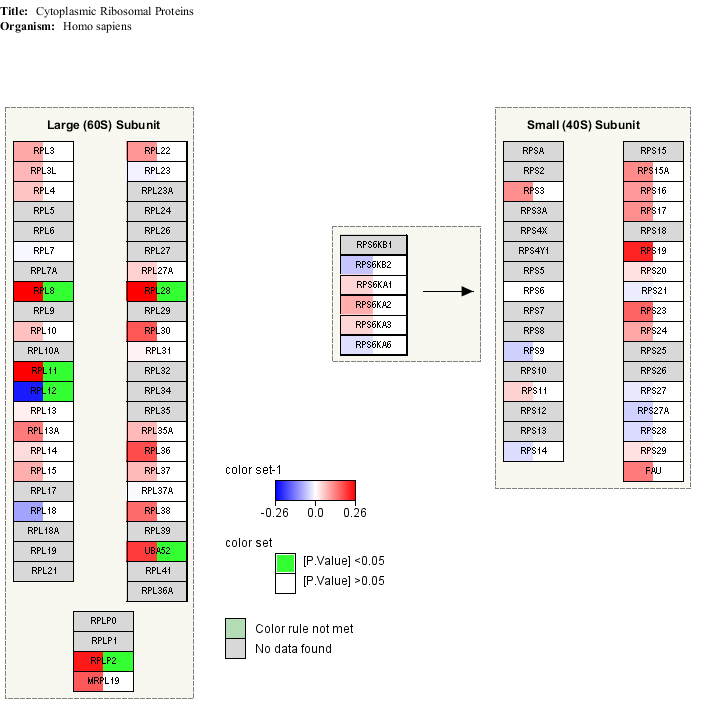


Figure 2. Harvie et al. Gene expression differences for the wikipathway ‘cytoplasmic ribosomal proteins’ in high versus low responders at baseline.


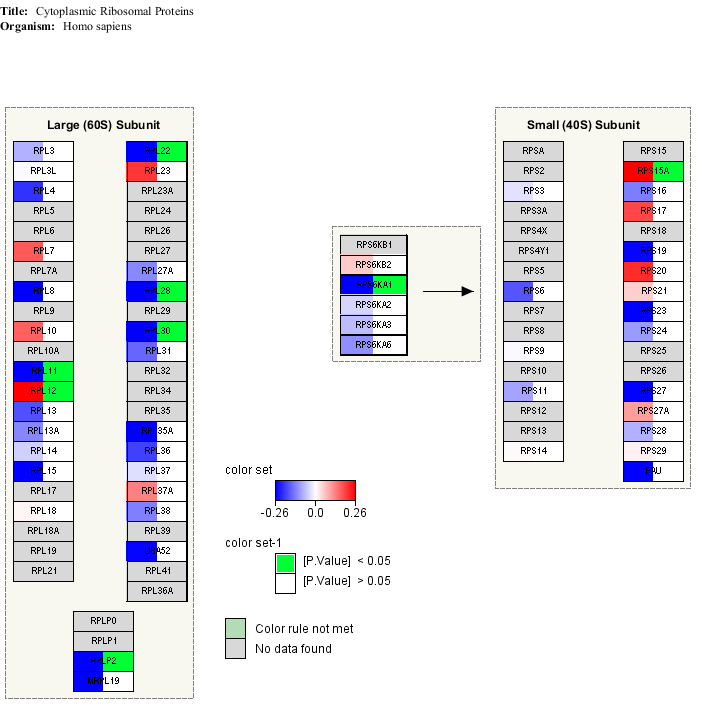


Figure 3. Rendo-Urteaga et al. Gene expression differences for the wikipathway ‘cytoplasmic ribosomal proteins’ in high versus low responders at baseline.


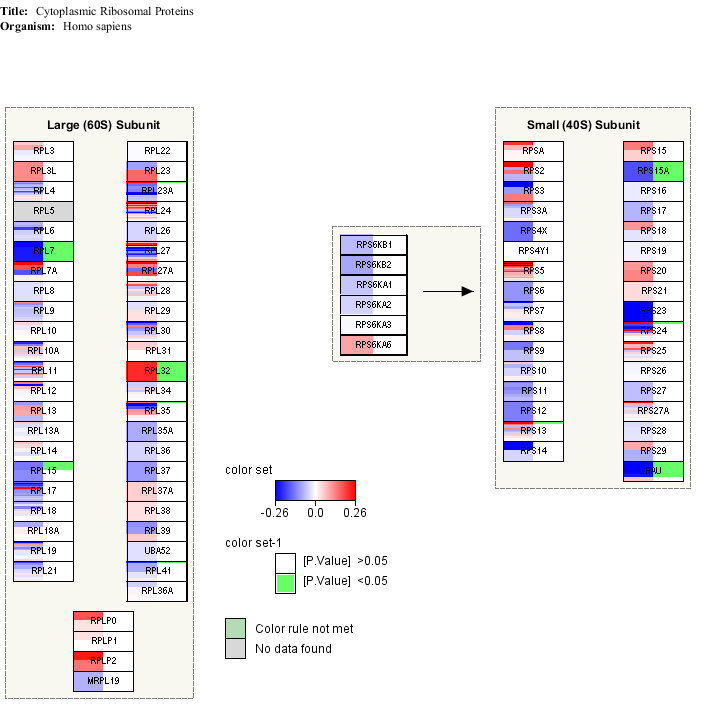

Supplement: Supplementary file 5 — Additional file 5: PathVisio diagrams of the pathway “cytoplasmic ribosomal proteins” for the comparisons in which this pathway was significantly enriches. Figure S1. Harvie et al. Gene expression differences for the wikipathway ‘cytoplasmic ribosomal proteins’ in baseline compared to post-intervention. Figure S2 Harvie et al. Gene expression differences for the wikipathway ‘cytoplasmic ribosomal proteins’ in high versus low responders at baseline. Figure S3. Rendo-Urteaga et al. Gene expression differences for the wikipathway ‘cytoplasmic ribosomal proteins’ in high versus low responders at baseline. [file 12263_2021_692_MOESM5_ESM.docx]
